# Anticipating implementation of colorectal cancer screening in The Netherlands: a nation wide survey on endoscopic supply and demand

**DOI:** 10.1186/1471-2407-12-46

**Published:** 2012-01-26

**Authors:** Sietze T van Turenhout, Jochim S Terhaar sive Droste, Gerrit A Meijer, Ad A Masclée, Chris JJ Mulder

**Affiliations:** 1 Department of Gastroenterology and Hepatology, VU University Medical Centre, P.O. Box 7057, 1007, MB Amsterdam, The Netherlands; 2 Pathology, VU University Medical Centre, Amsterdam, The Netherlands; 3 Division of Gastroenterology and Hepatology, Department of Internal Medicine, Maastricht University Medical Centre, Maastricht, The Netherlands; 4 Dutch Society of Gastroenterology, Haarlem, The Netherlands

**Keywords:** Endoscopy, Capacity, CRC screening, Colonoscopy, Endoscopist, Supply

## Abstract

**Background:**

Colorectal cancer (CRC) screening requires sufficient endoscopic resources. The present study aims to determine the Dutch endoscopic production and manpower for 2009, evaluate trends since 2004, determine additional workload which would be caused by implementation of a CRC screening program, and inventory colonoscopy rates performed in other European countries.

**Methods:**

All Dutch endoscopy units (N = 101) were surveyed for manpower and the numbers of endoscopy procedures performed in 2009. Based on calculations in the report issued by the Dutch Health Council, future additional workload caused by faecal immunochemical test (FIT) screening was estimated. The number of colonoscopies performed in Europe was evaluated by a literature search and an email-inquiry.

**Results:**

Compared to 2004, there was a 24% increase in total endoscopies (N = 505,226 in 2009), and a 64% increase in colonoscopies (N = 191,339 in 2009) in The Netherlands. The number of endoscopists had increased by 4.6% (N = 583 in 2009). Five years after stepwise implementation of FIT-based CRC screening, endoscopic capacity needs to be increased an additional 15%. A lack of published data on the number of endoscopies performed in Europe was found. Based on our email-inquiry, the number of colonoscopies per 100,000 inhabitants ranged from 126 to 3,031 in 15 European countries.

**Conclusions:**

Over the last years, endoscopic procedures increased markedly in The Netherlands without a corresponding increase in manpower. A FIT-based CRC screening program requires an estimated additional 15% increase in endoscopic procedures. It is very likely that current colonoscopy density varies widely across European countries.

## Background

The demand for gastrointestinal endoscopic procedures and gastroenterological care in The Netherlands and surrounding countries has been steadily increasing over the years [1]. Demographic changes due to an aging population and technical developments like video capsule endoscopy, double balloon endoscopy, and magnifying techniques, are likely to have contributed to this increase in workload. The existing shortage of gastroenterologists and endoscopy staff in The Netherlands is clear from waiting lists and vacancies [1]. This shortage is likely to be compounded by the implementation of a nationwide screening program for colorectal cancer (CRC), as it would lead to a further increase in endoscopic demand.

CRC screening programs have to comply with the Wilson and Jungner criteria including the availability of sufficient facilities for diagnosis and treatment [2]. Several European countries favour population-based screening by faecal occult blood tests, which has been shown to decrease CRC-related mortality [3-5]. Recently, the Dutch Minster of Health has decided to start the implementation of a CRC screening program in The Netherlands in 2013 [6]. In these program individuals aged 55-75 years are offered a faecal immunochemical test (FIT) biennially, at a cut-off value of ≥75 ng/ml [7]. All individuals with a positive FIT should subsequently be referred for colonoscopy.

To meet the Wilson and Jungner criteria, current endoscopic capacity in The Netherlands as well as other European countries needs to be determined. On behalf of the Dutch Society of Gastroenterology, this study aims to update current endoscopic production and determine the trends in endoscopic procedures performed in The Netherlands since 2004. In addition, the impact of implementing a FIT-based screening program on daily endoscopic practice is evaluated, and the number of colonoscopies currently being performed in several European countries is inventoried. In this study, a large increase in colonoscopies performed in The Netherlands is shown. For Europe, the number of colonoscopies performed appears to vary widely, but published data are lacking.

## Methods

### Study design

In spring 2010, a questionnaire was sent to all endoscopic facilities (N = 101) in The Netherlands. Respondents could provide their data by returning the questionnaire by mail, fax, email or by completing the query sheet on the internet by a link provided. Initial non-responders were sent reminders by email and mail. When needed, endoscopists were contacted by telephone.

The questionnaire concerned the endoscopic facilities in 2009, which were previously evaluated in 1999 and 2004 [1,8]. The total number of endoscopic procedures, as well as the number of gastroscopies, colonoscopies, sigmoidoscopies and endoscopic retrograde cholangiopancreatographies (ERCPs), were listed. Other topics evaluated the volume of nursing staff, length of the waiting list for elective gastroscopy and colonoscopy, and the number of endoscopists per hospital. Moreover, respondents were asked whether or not they expected that their endoscopy unit could cope with an increase of 30% in endoscopic workload in 2012. For comparison between 2004 and 2009, all paediatricians performing endoscopy were excluded in comparative analysis.

A literature search was carried out to determine the number of colonoscopies performed in surrounding European countries. Using PubMed, an electronic search was performed for manuscripts concerning endoscopic capacity published from 2000 to 2010. The terms used in the search were (gastrointestinal endoscopy AND supply), (gastrointestinal endoscopy AND capacity), (gastrointestinal endoscopy AND resource), (gastrointestinal endoscopy AND demand), (colonoscopy AND supply), (colonoscopy AND capacity), (colonoscopy AND resource), (colonoscopy AND demand). In addition, due to a lack of published data from European countries (see Results section), endoscopists associated with the United European Gastroenterology Federation were requested to report data on the number of colonoscopies per 100,000 inhabitants from their country, when available.

### Statistical analysis

At analyses, the number of endoscopic procedures was determined per 100,000 inhabitants. Geographical differences throughout the twelve provinces of The Netherlands were evaluated for endoscopic procedures, manpower and workload per endoscopist. Data on the age distribution of the Dutch population and the number of inhabitants per province were obtained from Statistics Netherlands [9]. Results were compared with data from 1999 to 2004 to assess the incremental trends in endoscopic procedures over the past five and ten years [1,8]. The report of the Dutch Health Council was used to study the impact of future CRC screening on daily practice. The Council has calculated that five years after a stepwise implementation of the screening program, 78,000 extra colonoscopies would be needed each year [7]. These calculations considered the size of the target screening population, an anticipated participation of 60% for FIT screening, and a FIT positivity rate of 6.4% at the proposed cut-off level of ≥75 ng/ml. Considering this report, the additional number of colonoscopies needed in a FIT-based national screening program was determined per region and endoscopy unit. SPSS for Windows Version 15.0 (SPSS Inc., Chicago, USA) was used for descriptive analyses.

## Results

### Response rate

The response rate to the Dutch survey was 100%, covering 48,908 hospital beds. There were no missing data concerning the total number of endoscopies, which included the number of gastroscopies, colonoscopies, sigmoidoscopies and ERCPs. Regarding the number of endoscopists at each site, 1 respondent did not provide information on the number of gastroenterologists, 2 respondents did not provide information on the number of internists and 4 respondents did not provide information on the number of surgeons performing endoscopies. Therefore, data on the number of gastroenterologists, internists and surgeons performing endoscopy were complete in 99%, 99% and 96%, respectively.

### Number of endoscopists

The total number of gastroenterologists, internists and surgeons performing endoscopies was 324, 256 and 103, respectively. The comparison with the number of endoscopists in 2004 is shown in Table 1. For gastroenterologists, a 47% increase over 2004 was found, whereas the number of internists and surgeons decreased by 27% and 16%, respectively. In total, a 4.6% increase in the number of endoscopists was found over the past five years. The total Dutch population increased by 1.4% in the same period [9].

**Table 1:** Number of gastroenterologists, internists and surgeons performing endoscopies in The Netherlands in 2004 and 2009

Endoscopists	2004	2009	Change (CI)
Gastroenterologists	221	324	+46.6% (40-53)

Internists	213	156	-26.8% (21-33)

Surgeons	123	103	-16.3% (12-26)

**Total**	**557**	**583**	**+4.6% (3-6)**

*CI *confidence interval

### Number of endoscopies

In 2009, 505,226 endoscopies were performed in The Netherlands, representing an increase of 24% compared to 2004 (N = 408,982) and 55% compared to 1999 (N = 325,000). The majority of the endoscopic procedures were gastroscopies (43%) and colonoscopies (38%; see Table 2). The number of colonoscopies increased substantially (64%) compared with 2004 (N = 191,339 in 2009 versus N = 116,815 in 2004), whereas the number of sigmoidoscopies decreased by 17% (N = 57,894 in 2009 versus N = 70,049 in 2004; see Table 2). This is further reflected by the increased ratio of colonoscopies to sigmoidoscopies (3.3: 1 for 2009 versus 1.7: 1 for 2004). The numbers of endoscopic procedures per 100,000 inhabitants are shown in Table 2.

**Table 2:** Number of endoscopic procedures in The Netherlands in 2004 and 2009; in total and per 100,000 inhabitants*

	Total number 2004	Total number 2009	Change	Endoscopies per 100,000 inhabitants 2004	Endoscopies per 100,000 inhabitants 2009	Change
Gastroscopies	184,915	216,267	+17.0%	1,137	1,312	+15.4%

Colonoscopies	116,815	191,339	+63.8%	719	1,161	+61.4%

Sigmoidoscopies	70,049	57,894	-17.4%	431	351	-18.5%

ERCPs	14,596	16,728	+14.6%	90	101	+12.7%

Others**	22,607	22,998	+1.7%	139	140	+0.4%

**Total**	**408,982**	**505,226**	**+23.5%**	**2,516**	**3,065**	**+21.8%**

*Dutch population 2004 and 2009: 16,258,032 and 16,485,787 respectively (www.cbs.nl). **Other endoscopic procedures, i.e. placement of duodenal feeding tubes, gastrostomies, paediatric endoscopies, double balloon enteroscopies, endoscopic ultrasound imaging and emergency procedures for haematemesis or haematochezia outside the endoscopic facility.

*ERCP *Endoscopic retrograde cholangiopancreatography

### Waiting list

The mean waiting time for elective gastroscopy and colonoscopy was 3.47 weeks (SD 2.66, range 0-13) and 4.79 weeks (SD 3.12, range 0-13), respectively. Compared with 2004, the waiting time increased for gastroscopy, but decreased for colonoscopy (3.0 and 5.1 weeks in 2004, respectively). Of all respondents, 22.4% expected their unit to be capable of handling a 30% increase in endoscopic workload in 2012.

### Geographical distribution

The number of endoscopists per 100,000 individuals ranged from 2.87 in Flevoland to 4.28 in Limburg. In 5 provinces the number of endoscopists decreased over the past five years (up to a 13.3% decrease), whereas in the other 7 the number increased (up to a 22.2% increase). In general, the number of endoscopies performed per endoscopist increased compared to 2004 (mean 18.1%, range 1.1-34.1%; see Table 3).

**Table 3:** Change in the number of endoscopists and the number of endoscopies performed per endoscopist over the twelve provinces of The Netherlands from 2004 to 2009

Province	Number of endoscopists 2004	Number of endoscopists 2009	Change	Endoscopies/endoscopist 2004	Endoscopies/endoscopist 2009	Change
Noord-Holland	92	98	+6.5%	678	841	+24.1%

Zuid-Holland	129	126	-2.3%	718	917	+28.3%

Noord-Brabant	65	76	+16.9%	787	883	+12.2%

Utrecht	43	40	-7.0%	812	873	+7.5%

Gelderland	65	71	+9.2%	726	769	+6.0%

Overijssel	32	35	+9.4%	998	1,158	+16.1%

Groningen	26	23	-3.1%	760	1014	+33.4%

Flevoland	9	11	+22.2%	632	847	+34.1%

Zeeland	15	13	-13.3%	602	742	+23.2%

Limburg	41	48	+17.1%	726	817	+12.5%

Friesland	24	23	-4.2%	620	733	+18.2%

Drenthe	16	19	+18.8%	637	644	+1.1%

The Netherlands	**557**	**583**	**+4.6%**	**734**	**867**	**+18.1%**

**Figure 1:**
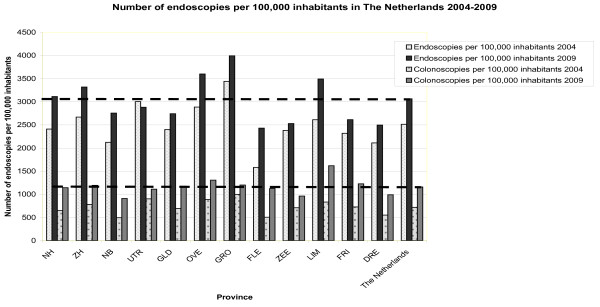
**Geographical distribution of the number of endoscopies and colonoscopies performed per 100,000 inhabitants in The Netherlands in 2004 and 2009**. Discontinous lines represent mean of The Netherlands. *NH *Noord-Holland, *ZH *Zuid-Holland, *NB *Noord-Brabant, *UTR *Utrecht, *GLD *Gelderland, *GRO *Groningen, *FLE *Flevoland, *ZEE *Zeeland, *LIM *Limburg, *FRI *Friesland, *DRE *Drenthe.

In all but one province, the total number of endoscopies performed in 2009 has increased compared with 2004 (mean 23.6%, range 0-64%, see Table 4). Per 100,000 inhabitants, the fewest endoscopies were performed in Flevoland (N = 2,431), and the most were performed in Groningen (N = 3,993). The number of endoscopies performed per 100,000 inhabitants in each region showed a mean increase of 21.6%. Only for Utrecht, the number of endoscopies per 100,000 inhabitants decreased (-4.0%), whereas in all other regions an increase was found. Flevoland was found to have the greatest increase of 53.8% (see Figure 1).

**Table 4:** Geographical distribution of the number of endoscopies in Dutch provinces in 2004 and 2009

Province	Number of endoscopies 2004	Number of endoscopies 2009	Change	Number of endoscopies per 100,000 inhabitants 2004	Number of endoscopies per 100,000 inhabitants 2009	Change
Noord-Holland	62,359	82,401	+32%	2,410	3,114	+29%

Zuid-Holland	92,172	115,541	+25%	2,670	3,319	+24%

Noord-Brabant	51,128	67,070	+31%	2,124	2,755	+30%

Utrecht	34,905	34,910	0%	3,003	2,883	-4%

Gelderland	47,181	54,604	+16%	2,399	2,742	+14%

Overijssel	31,921	40,518	+27%	2,887	3,600	+25%

Groningen	19,767	22,924	+16%	3,441	3,993	+16%

Flevoland	5,687	9,320	+64%	1,580	2,431	+54%

Zeeland	9,026	9,640	+7%	2,381	2,530	+6%

Limburg	29,767	39,205	+32%	2,613	3,492	+34%

Friesland	14,880	16,861	+13%	2,318	2,615	+13%

Drenthe	10,189	12,232	+20%	2,112	2,497	+18%

The Netherlands	**408,982**	**505,226**	**+24%**	**2,514**	**3,065**	**+22%**

In all provinces the number of colonoscopies increased substantially (mean 63.8%) with a range of 28% in Utrecht to 137% in Flevoland (see Table 5). Geographical distribution of the number of colonoscopies per 100,000 individuals ranged from 911 in Noord-Brabant to 1,620 in Limburg (mean in The Netherlands was 1,164). The increase of the number of colonoscopies per 100,000 inhabitants ranged from 20.3% in Groningen to 122.6% in Flevoland (mean increase 64.4%; see Figure 1). Also the mean number of colonoscopies performed per endoscopist increased substantially from 210 in 2004 to 328 in 2009 (mean increase of 56.6%; range 37.2-94.0%; see Table 3). The most colonoscopies per endoscopist were performed in Overijssel (N = 420) and the fewest colonoscopies per endoscopist were performed in Drenthe (N = 256).

**Table 5:** Geographical distribution of the number of colonoscopies in Dutch provinces in 2004 and 2009

Province	Number of colonoscopies 2004	Number of colonoscopies 2009	Change	Number of colonoscopies per 100,000 inhabitants 2004	Number of colonoscopies per 100,000 inhabitants 2009	Change
Noord-Holland	16,798	30,281	+80%	649	1,144	+76%

Zuid-Holland	26,992	41,583	+54%	782	1,194	+53%

Noord-Brabant	11,932	22,191	+86%	496	911	+84%

Utrecht	10,498	13,473	+28%	903	1,113	+23%

Gelderland	13,716	23,248	+70%	697	1,168	+68%

Overijssel	9,799	14,709	+50%	886	1,307	+48%

Groningen	5,739	6,906	+20%	999	1,203	+20%

Flevoland	1,822	4,319	+137%	506	1,126	+123%

Zeeland	2,691	3,670	+36%	710	963	+36%

Limburg	9,487	18,184	+92%	833	1,620	+94%

Friesland	4,680	7,918	+69%	729	1,228	+68%

Drenthe	2,661	4,857	+83%	552	991	+80%

The Netherlands	**116,815**	**191,339**	**+64%**	**719**	**1,161**	**+61%**

### Number of colonoscopies in Europe

The literature search yielded data on endoscopic procedures only for Ireland and Romania [10,11]. Data for Italy were obtained by extrapolation of a recent, prospective Italian survey and Italian life tables [12,13]. Because of this disappointing result, dedicated endoscopists in several European countries were requested to provide the number of colonoscopies performed per 100,000 inhabitants in their country, when available. In 15 of the European countries that were contacted, the number of colonoscopies per 100,000 inhabitants was reported (see Table 6). For Spain and the United Kingdom, either no data or only the number of screening colonoscopies were known. The number of reported colonoscopies per 100,000 inhabitants ranged from less than 126 in Turkey to 3,031 in Germany. The number of colonoscopies performed in The Netherlands is similar to around half of other European countries (7 of 15 countries ranged 950-1,263).

**Table 6:** Overview of the number of colonoscopies performed per 100,000 inhabitants in different European countries

Country	Year of reference	Number of colonoscopies per 100,000 inhabitants	Remarks
Austria	2009	1,131	Excluding number of in hospital colonoscopies

Estonia	2008	992	

Finland	2010	950	Based on regional data (Helsinki and surrounding areas)

France	2008	1,837	

Germany	2009	3,031	Includes a 10% estimation for people with private insurance. All colonoscopies performed include 19% screening colonoscopies.

Ireland	2009	2,020	

Italy	2004	570	Extrapolation from references [12,13]

Latvia	2009	723	

Luxembourg	2008	2,889	Concerns colonoscopies and sigmoidoscopies

Norway	2006	1,060	More recent data expected shortly

The Netherlands	2009	1,161	

Romania	2009	178	

Poland	2010	1,263	Based on prescriptions for bowel preparation

Sweden	2010	1,100	Based on regional data (Stockholm area)

Turkey	+/- 2009	126	Concerns total of lower gastrointestinal endoscopies [14]

For Spain and the United Kingdom, no total data for the number of colonoscopies were available

### Anticipation of a future CRC screening program

The Dutch Health Council has reported that a stepwise implementation of a biennial FIT-based CRC screening program would require an additional 78,000 colonoscopies per year in year six of the screening program. These 78,000 extra colonoscopies represent a 15% increase based on current endoscopies performed, and a 41% increase based on current colonoscopies performed. This would result in a mean increase from the current 1,164 colonoscopies per 100,000 inhabitants to 1,634 colonoscopies per 100,000 inhabitants.

Based on the geographical distribution of inhabitants aged 55-75 in 2009 [9], the number of extra colonoscopies needed after five years of screening was calculated per province (range 1,379-15,774). The mean number of additional colonoscopies needed per endoscopy unit per year was 754 (range 460-1,029). Assuming that endoscopy services are running 46 weeks per year, the number of additional colonoscopies needed per unit per week and per day ranged from 10.0 to 22.4 and 2.0 to 4.5, respectively (see Table 7).

**Table 7:** Required number of extra colonoscopies after implementation of a biennial faecal immunochemical test based CRC screening program for subjects 55-75 years old

Province	Number of inhabitants 55-75 years old 2009 (% of The Netherlands)*	Number of extra colonoscopies needed	Number of extra colonoscopies needed per unit	Number of extra colonoscopies needed per unit per week	Number of extra colonoscopies needed per unit per day
Noord-Holland	538,936 (16%)	12,104	712	15,5	3,1

Zuid-Holland	702,358 (20%)	15,774	789	17,1	3,4

Noord-Brabant	534,288 (15%)	12,000	923	20,1	4,0

Utrecht	228,423 (7%)	5,130	733	15,9	3,2

Gelderland	428,848 (12%)	9,631	876	19,0	3,8

Overijssel	231,492 (7%)	5,199	867	18,8	3,8

Groningen	122,408 (4%)	2,749	687	14,9	3,0

Flevoland	61,387 (2%)	1,379	460	10,0	2,0

Zeeland	90,896 (3%)	2,041	680	14,8	3,0

Limburg	274,970 (8%)	6,176	1,029	22,4	4,5

Friesland	143,445 (4%)	3,222	644	14,0	2,8

Drenthe	115,547 (3%)	2,595	649	14,1	2,8

The Netherlands	3,472,998 (100%)	78,000	788	17,1	3,4

* www.cbs.nl*CRC *colorectal cancer

## Discussion

This nationwide endoscopy survey had a 100% response rate and has shown a 24% increase in endoscopies performed in The Netherlands over the past five years, and a 55% increase over the past 10 years. In particular, the number of colonoscopies increased by 64%. This is accompanied by only a minor increase in the number of endoscopists. Anticipating a national FIT-based screening program, an additional 10-22 colonoscopies per unit per week would be required in year six of the screening program, necessitating a 15% increase over the current endoscopic workload. Putting these figures into a European perspective, it was shown that almost half of the European countries that responded performed similar quantities of colonoscopies per 100,000 inhabitants. Yet, a striking heterogeneous distribution across Europe was observed.

An increase in the number of endoscopies was anticipated due to population growth and potentially changing morbidity patterns. However, the 64% increase in the number of colonoscopies over the past five years is remarkable. Several CRC screening trials have been ongoing in The Netherlands since 2009 [15-17], but the cumulative number of extra colonoscopies in these trials is estimated not to exceed 2,500. Consequently, these trials will only have a minor effect on the total number of colonoscopies. Due to the increasing awareness of CRC and the need for a screening program, opportunistic screening has probably increased as well. In addition, although speculative, considering sigmoidoscopy rates decreased over the same time period, subjects who present with rectal bleeding may be being referred for total colonoscopy more frequently.

Although the total number of endoscopists increased slightly, the number of gastroenterologists increased substantially whereas the number of internists and surgeons performing endoscopies in 2009 declined markedly. The increase in the total number of endoscopies performed might be related to this change as gastroenterologists might spend more full time equivalents on performing endoscopies, what could result in an increase in endoscopic production. However, full time equivalent volumes on endoscopic procedures per endoscopist's specialty is unknown.

The number of endoscopies performed varied substantially over the different provinces of The Netherlands. Yet, per 100,000 inhabitants, this difference was at maximum 1.6 fold. These differences might be related to variation in available endoscopists and, although speculative, morbidity patterns. A difference in patient demographics among provinces as a potential explanation, could not be found [9].

A more extensive interpretation of the presented results in the context of the number of endoscopies performed in other European countries is difficult. Although sufficient endoscopic resources are clearly stated as a prerequisite before implementing a CRC screening program in the recent European CRC screening guidelines [18], very little data on European production are available. Only from Ireland and Romania have recent data been published [10,11]. Other studies did not report on current and total capacity, but did report on models of capacity needed in CRC screening [19,20]. The results of our pilot inquiry indicate that the number of colonoscopies per 100,000 inhabitants has a wide range from less than 126 in Turkey to 3,031 in Germany. If Romania and Turkey are considered representatives of Eastern Europe, then endoscopic capacity in Eastern Europe needs to be increased [11]. Half of the responding countries perform 950-1,263 colonoscopies per 100,000 inhabitants.

Anticipating a national screening program in The Netherlands, current capacity may be insufficient as the number of extra colonoscopies needed is expected to range from 10 to 22 per unit per week. Although subjective, 22% of endoscopy units expect to be able to cope with a 30% increase in workload in 2012, whereas the anticipated increase would only be 15%. The number of endoscopic procedures performed in 2009 are considered current minimal capacity. However, capacity also relies on endoscopy staff, medical co-workers, flexible endoscopes, (desinfecting) material, medication and effective use of resources. In addition, the number of endoscopies is also influenced by procedure guidelines and quality issues. For example, in 2004 the guideline for cleansing and desinfection of flexible endoscopes was intensified [21]. Current available facilities in Dutch endoscopy units are unknown. Improvements in operational efficiency and/or technological advancements could increase current capacity. Rest capacity is however unknown and warrants additional studies. Therefore, current capacity level may actually be higher or lower than the production level. Still, there might be no or little rest capacity as for years many vacancies for gastroenterologist exists in The Netherlands [22]. It can be expected that capacity needs to increase at least to some extent, as more colonoscopies need to be performed in a screening program. Also, an increase in workload for medical co-workers can be expected as e.g. in upper gastrointestinal endoscopies, it was shown that around 75% of total time was spent on pre-and postendoscopic operations [23]. Importantly, before implementing screening based on the present results, it should be ascertained that the increase in procedures performed does not hamper quality of the procedures. Due to the substantial increase over the recent years without a considerable increase in manpower, the upper limit of capacity might have been reached already, and a further increase without sufficient investment might result in low quality colonoscopies.

Whether investment in increasing capacity is needed or not, eventually the number of endoscopic procedures will decrease again. It is estimated that 10% of colonoscopies performed in daily practice are performed for opportunistic screening [24], which will become unnecessary after the implementation of a national screening program. In addition, less colonoscopies due to less frequent symptomatic presentations of CRC and a decrease in CRC incidence is expected [25-28]. On the contrary, the implementation of a screening program will also result in more adenomas being detected, that consequently warrants ongoing colonoscopic surveillance. Capacity could be increased by critically reviewing guidelines for post-polypectomy surveillance [29,30].

Some limitations of the present study need to be discussed. Firstly, this study describes the changes in daily endoscopic practice, but the factors that drive these changes are largely unknown. Secondly, due to missing data on full time equivalents for 2004, the trends in fulltime manpower could not be determined completely. A substantial increase in the number of endoscopies performed per endoscopist was found, so even if more endoscopists would work part-time in 2009, this number would in fact be even higher. Thirdly, the response rate for the data from 2004 was slightly lower than for 2009 (98% versus 100% respectively). Therefore, the increases presented could be slight overestimations and the decreases could be slight underestimations. However, modelling the missing data based on the complete data of 2009, the over/underestimation is expected not to be larger than 1-3% (data not shown). Fourthly, it was assumed that the 78,000 extra colonoscopies required for FIT-based CRC screening would distribute according to the number of inhabitants aged 55-75 in the different regions of The Netherlands, and that all individuals would get their colonoscopy in the province of residence. However, participation rates for FIT screening might show regional differences, and patients may prefer the closest hospital instead of a hospital in their own province. Fifth, the data presented for other European countries should be interpreted with caution. The data provided by European colleagues were mainly obtained from national or regional registries. As these data were not collected in the same standardized manner and therefore were not validated by the authors of this manuscript, the European data may not be the exact reflection of each country. Still, these data are provided with discretion by the European endoscopists, and we believe this effort is a good first inventory of current endoscopic variety. In addition, the lack of published data warrants more studies especially from countries in which CRC screening is advocated, planned or already implemented.

## Conclusions

In conclusion, current Dutch endoscopic capacity has increased substantially since 2004. For the number of colonoscopies in particular, a remarkable increase was found, although the number of endoscopists increased marginally. When a national CRC screening program will be implemented in The Netherlands, the increase of colonoscopies needed per unit will be at maximum 22 per week. Finally, the distribution of colonoscopies performed in Europe is very heterogeneous and more solid data are needed for proper analysis.

## Competing interests

The authors declare that they have no competing interests. S.T. van Turenhout was supported by a research grand from CTMM (Centre for Translational Molecular Medicine), The Netherlands. This company had no influence on any aspect relevant to this study.

## Authors' contributions

STVT and JSTSD participated in the study concept and design; acquisition of data; analysis and interpretation of data, statistical analysis and drafting of the manuscript. GAM and AAM participated in the study concept and design; acquisition of data and critical revision of the manuscript for important intellectual content. CJJM participated in the study concept and design; acquisition of data; analysis and interpretation of data, critical revision of the manuscript for important intellectual content, and study supervision. All authors read and approved the final manuscript.

## Pre-publication history

The pre-publication history for this paper can be accessed here:

http://www.biomedcentral.com/1471-2407/12/46/prepub
